# Concern with reproducibility of rehabilitation after critical illness and hospital discharge

**DOI:** 10.1186/s13054-021-03745-9

**Published:** 2021-09-15

**Authors:** Kota Yamauchi, Shunsuke Kina, Shunsuke Taito

**Affiliations:** 1 Department of Rehabilitation, Steel Memorial Yawata Hospital, Harunomachi 1-1-1, Yahatahigashi-ku, Kitakyushu, Fukuoka 805-8508 Japan; 2 Department of Rehabilitation, Nakagami Hospital, Noborikawai 610, Okinawa, 904-2195 Japan; 3 Division of Rehabilitation, Department of Clinical Practice and Support, Hiroshima University Hospital, Kasumi 1-2-3, Minami-ku, Hiroshima, 734-8551 Japan; 4 Systematic Review Workshop Peer Support Group (SRWS-PSG), Osaka, Japan

**Keywords:** Post-intensive care syndrome, Critical illness, Rehabilitation, Return to work

To the editor,

We read with great interest the article by Major and colleagues, who examined the feasibility of an interdisciplinary rehabilitation program designed for patients with physical impairment of post-intensive care syndrome (PICS) who are discharged home [1]. However, we would like to highlight two concerns regarding this study.

First, the intervention of the REACH study was highly feasible and led to higher patient satisfaction; however, it is unclear how the intervention differs from usual care (UC). All patients in the intervention group received physical therapy (PT), but after 3 months, one-third of the intervention group had completed PT. The frequency of intervention was higher in UC, which is presumed to be based on the Dutch practical recommendation for PT [2]. In addition, the intervention rate of occupational therapy (OT) increased 2.5 times in 3–6 months compared to 0–3 months, showing an increase in contrast to PT. If more detailed information is provided regarding the termination criteria for PT and OT for intervention groups, it will be easier to reproduce high feasibility and patient satisfaction for patients with PICS.

Second, the high return to work (RTW) in the REACH study might be due to failure to return to the same job prior to the disease onset. The problem with RTW for critical illness survivors is the imbalance between the job workload and functional ability [3]. A recent review reported a 5%–84% worsening employment status and 17–66% occupation change in previously employed survivors of critical illness [4]. If the percentage of patients in each group returning to the same work as before the onset and the time to RTW is indicated, readers can understand how to support interdisciplinary collaboration and highly satisfied reinstatement.

Elucidating the aforementioned factors might help in better interpretation of the results and establish a better home-based interdisciplinary rehabilitation program for physical impairment from PICS.

## Data Availability

Not applicable.

## References

[1] Major ME, Dettling-Ihnenfeldt D, Ramaekers SPJ, Engelbert RHH, van der Schaaf M (2021). Feasibility of a home-based interdisciplinary rehabilitation program for patients with Post-Intensive Care Syndrome: the REACH study. Crit Care.

[2] Kwakman RCH, Major ME, Dettling-Ihnenfeldt DS, Nollet F, Engelbert RHH, van der Schaaf M (2020). Physiotherapy treatment approaches for survivors of critical illness: a proposal from a Delphi study. Physiother Theor Pract.

[3] Su H, Hopkins RO, Kamdar BB, May S, Dinglas VD, Johnson KL et al. Association of imbalance between job workload and functional ability with return to work in ARDS survivors. Thorax. 2021 :thoraxjnl-2020-21658610.1136/thoraxjnl-2020-21658633927021

[4] Kamdar BB, Suri R, Suchyta MR, Digrande KF, Sherwood KD, Colantuoni E (2020). Return to work after critical illness: a systematic review and meta-analysis. Thorax.

